# Cyclosporine A in Ullrich Congenital Muscular Dystrophy: Long-Term Results

**DOI:** 10.1155/2011/139194

**Published:** 2011-10-17

**Authors:** Luciano Merlini, Patrizia Sabatelli, Annarita Armaroli, Saverio Gnudi, Alessia Angelin, Paolo Grumati, Maria Elena Michelini, Andrea Franchella, Francesca Gualandi, Enrico Bertini, Nadir Mario Maraldi, Alessandra Ferlini, Paolo Bonaldo, Paolo Bernardi

**Affiliations:** ^1^Section of Medical Genetics, Department of Experimental and Diagnostic Medicine, University of Ferrara, 44121 Ferrara, Italy; ^2^Laboratorio Di Biologia Cellulare Muscoloscheletrica, Istituto Ortopedico Rizzoli, 40136 Bologna, Italy; ^3^Istituto di Genetica Molecolare, CNR, Istituto Ortopedico Rizzoli, 40136 Bologna, Italy; ^4^Medicina Generale, Istituto Ortopedico Rizzoli, 40136 Bologna, Italy; ^5^Department of Biomedical Sciences, University of Padova, 35121 Padova, Italy; ^6^Department of Histology, Microbiology and Medical Biotechnology, University of Padova, 35121 Padova, Italy; ^7^Pediatric Surgery Department, Sant'Anna Hospital, Corso Giovecca 203, 44121 Ferrara, Italy; ^8^Unit for Neuromuscular Disorders, Department of Neurosciences and Laboratory of Molecular Medicine, Bambino Gesu' Children's Research Hospital, 00165 Roma, Italy

## Abstract

Six individuals with Ullrich congenital muscular dystrophy (UCMD) and mutations in the genes-encoding collagen VI, aging 5–9, received 3–5 mg/kg of cyclosporine A (CsA) daily for 1 to 3.2 years. The primary outcome measure was the muscle strength evaluated with a myometer and expressed as megalimbs. The megalimbs score showed significant improvement (*P* = 0.01) in 5 of the 6 patients. Motor function did not change. Respiratory function deteriorated in all. CsA treatment corrected mitochondrial dysfunction, increased muscle regeneration, and decreased the number of apoptotic nuclei. Results from this study demonstrate that long-term treatment with CsA ameliorates performance in the limbs, but not in the respiratory muscles of UCMD patients, and that it is well tolerated. These results suggest considering a trial of CsA or nonimmunosuppressive cyclosporins, that retains the PTP-desensitizing properties of CsA, as early as possible in UCMD patients when diaphragm is less compromised.

## 1. Introduction

Collagen VI (ColVI) is an ubiquitously expressed extracellular matrix (ECM) protein consisting of three chains, alpha1(VI), alpha2(VI), and alpha3(VI), encoded by the *COL6A1*, *COL6A2*, and *COL6A3* genes, respectively. Mutations in these three genes result in two major clinical forms, Bethlem myopathy (BM [MIM158810]) [1] and Ullrich congenital muscular dystrophy (UCMD [MIM 254090]) [2], and in the rarer myosclerosis myopathy (MM [MIM 255600]) [3]. BM is characterized by slowly progressive axial and proximal muscle weakness with finger flexion contractures [4]. BM is usually mild [4], sometimes slowly progressive with some affected individuals over 50 years of age needing aids for outdoors mobility [5]. In contrast, UCMD is a severe disorder characterized by congenital muscle weakness with axial and proximal joint contractures and distal joint hypermobility [6]. Motor milestones are delayed, and most of the children never acquire the ability to walk independently [6]. Early, progressive and severe respiratory involvement may require artificial ventilatory support in the first or second decade of life [7]. Both recessive and dominant ColVI mutations lead to BM and UCMD [8, 9]. Prevalence has been estimated as 0.5 : 100,000 for BM and 0.1 : 100,000 for UCMD [10], but these disorders are probably underdiagnosed.

In the *Col6a1^−/−^* mutant mouse [11], muscle fibres have a loss of contractile strength associated with ultrastructural alterations consisting of marked dilations of the sarcoplasmic reticulum, mitochondrial alterations, and nuclear features of apoptosis [12]. Muscle fibers of *Col6a1^−/−^* mice show in addition signs of mitochondrial dysfunction. The resting mitochondrial membrane potential is unaffected, but addition of oligomycin causes depolarization, an abnormal response that is prevented by plating fibers on ColVI or by treating them with cyclosporin A (CsA) [12], a drug which desensitizes the mitochondrial permeability transition pore through binding to cyclophilin D [13]. In order to establish whether mitochondria are also involved in the pathogenesis of UCMD and BM, we studied muscle biopsies and myoblasts from patients [14]. UCMD patients displayed an increased rate of apoptosis in skeletal muscle *in vivo* and in primary myoblast cultures [14]. The latter also displayed a measurable fraction of altered mitochondria (with morphological alterations ranging from shape changes to overt swelling) and a *latent mitochondrial dysfunction* that could be revealed by the addition of oligomycin, which caused depolarization only in mitochondria from patients [14]. In an open trial with CsA, we enrolled five patients representing the clinical and molecular variety of ColVI myopathies [15]. Muscle biopsy was done before the onset of treatment and mitochondria within the cells isolated from patients depolarized after the addition of oligomycin, confirming *in vivo *the presence of the latent dysfunction previously identified in *Col6a1^−/−^*  mice and in cultures from UCMD patients [14]. One month after oral administration of CsA at a dose of 5 mg/kg per day in two divided doses, a new biopsy was taken at the contralateral site, and the experiment was repeated [15]. Strikingly, the mitochondrial membrane potential response to oligomycin was largely normalized in the muscle cells from all patients, indicating that at the dose used in this study, CsA reaches pharmacologically active concentrations in muscle. In addition, unlike samples from normal donors where apoptosis was undetectable, all samples from patients had a sizeable number of apoptotic nuclei in the first biopsy, and treatment with CsA considerably decreased the occurrence of apoptosis in all patients, indicating a cause-effect relationship between normalization of mitochondrial function and decreased cell death. After CsA treatment, an increased number of regenerating myofibers were detected and particularly prominent in the three younger patients included in the study. 

At the end of the one-month pilot trial treatment, the parents of the three children requested to continue CsA treatment. In addition, we were able to recruit three more children with UCMD. Objective of this study is to evaluate the efficacy of a long-term treatment with CsA in six UCMD patients. 

## 2. Methods

### 2.1. Study Population

Six patients with Ullrich congenital muscular dystrophy (UCMD), three originally enrolled in the one-month pilot trial [15] and three new, received CsA treatment.

### 2.2. Trial Design

This is a prospective long-term, open, noncomparative, pilot clinical trial on the efficacy of CsA in patients with UCMD. The trial design conforms the EMEA guidelines [16] for clinical trials in very rare diseases.

### 2.3. Efficacy Measurements

The primary endpoint of the trial was an increase in muscle strength as measured with hand-held dynamometer. The secondary endpoints were the effect of CsA on the change in muscle mass, in motor function, and in respiratory function in patients with UCMD. In two of the new patients, the biological endpoint was the evaluation of biomarkers of mitochondrial function, apoptosis, and regeneration at baseline and after 3 months of CsA treatment.

Muscle strength was measured by maximum voluntary isometric contraction, with a hand-held dynamometer (Cit Technics, Groningen, The Netherlands) to determine the megalimbs, a composite score resulting from the sum of bilateral muscle strength of elbow flexion, hand grip, knee flexion, and knee extension. Measurements obtained by standard techniques and in standard position were repeated three times, and the best score was noted [17]. We have already provided evidence of reliability and sensitivity of this technique [17, 18], which we have employed in clinical trials [19].

The forced vital capacity was measured by a standard breathing test with a spirometer.

The 6-minute walking distance test was performed in the two patients able to walk [20]. 

The motor function was evaluated with the Muscular Dystrophy Functional Grading Scale for functional evaluation [21].

Muscle mass and body fat were evaluated by dual-energy X-ray densitometer (DXA).

Measurements of body composition were performed with the Norland XR-36 DXA (Norland, Fort Atkinson, Wis) with the subject supine. DXA estimates of body composition are based on a three-compartment model measuring bone mineral content (BMC), fat tissue mass (FTM), and lean tissue mass (LTM). Total body bone mineral content (BMD) is given as a *Z* score to compare with the normal range for children of the same age [22]. Body fat was also estimated with the body mass index (BMI) based on an individual's weight and height (wt/ht^2^). Different from adult, to interpret BMI in children, age and sex should be taken into account given that the amount of body fat changes with age and differs between girls and boys [23]. The CDC BMI-for-age growth charts for girls and boys take into account these differences and allow translation of a BMI number into a percentile for a child's or teen's sex and age [23].

We obtained muscle biopsies from healthy donors and patients after informed consent and approval of the Ethics Committee of the University of Ferrara. Muscle biopsy of the tibialis anterior was performed before and after one month of treatment in the first three patients [15] and after 3 months of full dose treatment in the last two patients. No muscle biopsy was done in patient 4, sibling of patient 2, in whom the positive effect of CsA *in vivo* was already shown. 

The preparations of the muscle biopsy for cell culture and immunocytochemistry have been extensively described [15]. In particular, early regenerating fibers [24–27] were detected by immunofluorescence analysis with antidevelopmental myosin heavy chain (dMHC) (Novocastra clone RNMy2/9D2, which recognize a myosin heavy chain embryonic form), and desmin antibodies [15], and necrosis with histochemical analysis of acid phosphatase and immunohistochemical detection of complement C5b-9 deposition within muscle fibers [28]. For statistical analysis of regeneration, 1,000 fibers from three different regions of the biopsy for each sample were considered.

### 2.4. Detection of Apoptosis

We measured the rate of apoptosis in muscle biopsies using the TUNEL method [15]. Seven-micrometer-thick frozen sections were prepared from muscle biopsies, fixed in 50% acetone/50% methanol, and processed for TUNEL analysis by using the Apoptag peroxidase *in situ *apoptosis detection kit (Chemicon). Visualization of all nuclei was performed by staining with Hoechst 33258 (Sigma). The number of TUNEL-positive nuclei was determined in randomly selected fields with a Zeiss Axioplan microscope (×40 magnification) equipped with a digital camera. For each biopsy, 30–85 fields containing muscle fibers and covering a total area of *≅*1–7 mm^2^ were observed.

### 2.5. Mitochondrial Membrane Potential Assay

Mitochondrial membrane potential was measured based on the accumulation of TMRM (Molecular Probes) [29]. Primary cell cultures obtained from patient biopsies were plated within 4 h of the surgical biopsy in complete medium plus 20% FCS on laminin/poly-L-lysine-coated coverslips (BD Laboratories), and allowed to attach and spread for 9–14 h. The medium was then replaced with serum-free DMEM supplemented with 10 nM TMRM for 30 min, and cellular fluorescence images were acquired with an Olympus IX71/IX51 inverted microscope, equipped with a xenon light source (75 W) for epifluorescence illumination and with a 12-bit digital cooled CCD camera (Micromax, Princeton Instruments). Data were acquired and analyzed using Cell R Software (Olympus). For detection of fluorescence, 568 ± 25 nm band-pass excitation and 585 nm long-pass emission filter settings were used. Images were collected with exposure time of 100 msec using a 40x, 1.3 NA oil immersion objective (Nikon) for the patients' cells. The extent of cell and hence mitochondrial loading with potentiometric probes is affected by the activity of the plasma membrane multidrug resistance pump, which is inhibited by CsA. Treatment with this drug may therefore cause an increased mitochondrial fluorescence that can be erroneously interpreted as an increase of the mitochondrial membrane potential. In order to prevent this artifact and to normalize the loading conditions, in all experiments with TMRM the medium was supplemented with 1.6 *μ*M CsH, which inhibits the multidrug resistance pump, but not the PTP [30]. At the end of each experiment, mitochondria were fully depolarized by the addition of 4 *μ*M of the protonophore carbonyl cyanide-*p*-trifluoromethoxy-phenyl hydrazone. Clusters of several mitochondria were identified as regions of interest, and fields not containing cells were taken as the background. Sequential digital images were acquired every 2 min.

### 2.6. Study Drug

CsA (sandimmun neoral) was given at the dosage of 5 mg/kg/die for one month [15] in the first 4 patients and for 3 months in the last two patients. After the initial period (1–3 months), a progressive tapering was planned to perform long-term low-dose (1.25–3 mg/kg/die) maintenance therapy (targeting blood concentration >200 ng/mL 2 h afterdosing). The daily dose was taken in two divided doses (morning and evening), after meals, dissolved in a drink. Serum concentration of CsA was monitored to adapt the therapeutic dosage with a target C2 levels in the range of 250–500 ng/mL [31, 32]. CsA-induced nephrotoxicity rarely develops using doses lower than 5 mg/kg/day, and it is generally reversible upon timely decrease of the dose of CsA or its discontinuation if the serum creatinine levels increase >25% above patients' baseline values [31]. Blood CsA levels, predose and two h afterdose, were determined with a no-pretreatment monoclonal antibody-based immunoassay, affinity-column-mediated immunoassay (ACMIA) using Dimension Xpand-HM for the measurement of CsA in whole blood.

### 2.7. Safety Evaluation

Side effects were assessed using an adverse events checklist, a complete blood count, and hepatic and renal function tests, done at baseline and every 1–3 months.

### 2.8. Data Analysis

Data were analyzed with the unpaired Students *t-*test, and values with *P* < 0.05 were considered as significant. Descriptive analysis was computed for each patient and for all patients. Continuous variables were presented as mean.

## 3. Results

The features of the patients are summarized in Table 1. ColVI levels are based on immunoistochemistry as described in original references [2, 14], which also contain further details about the gene mutations and their mode of inheritance. The three patients enrolled in the original pilot trial (P1, P2, and P3) received CsA for 2 to 3.2 years and the three new patients for 1 to 1.2 years (Table 2). The patients with UCMD had a very low residual muscle strength at baseline, 11% of the predicted in age-/sex- matched population [33] (Table 2). The megalimbs moved from 163.8 N to 195.3 N in the six patients with a 19% mean increase (*P* value is 0.0597, considered not quite significant; *t* = 2.425 with 5 degrees of freedom) (Table 2). Patient 5 had already an evident progression of the disorder before starting treatment. He was never able to run and lost the ability to rise from the floor at the age of 6 and to climb stairs at 7. His total body fat was 49% at age 6.5 (lean mass 12.7 kg), 70% at age 9.1 (lean mass 8.6 kg) when he started the treatment, and 79% (lean mass 7.1 kg) at the last followup one year later (Table 3). If we consider P5 a nonresponder, in the other five patients, the megalimbs increased from 148.4 N to 190.0 N with a 28% mean increase (*P* value is 0.0142, considered significant; *t* = 4.159 with 4 degrees of freedom). 

Motor function as assessed with the Muscular Dystrophy Functional Grading Scale remained the same in all the patients during the treatment period.

Respiratory function declined during the treatment (Table 2). Apart from patient 3, who was not able to perform spirometry because of limited bite opening, the other five patients showed a steady decline (−7% per year) of the % predicted forced vital capacity during the treatment period. The two oldest patients (P1 and P2), who at the start of treatment had already a FVC% of predicted below 50%, went down to 24% and 32%, respectively and showed marked oxygen desaturation at night. At this point (age 13 and 12), CsA treatment was interrupted and they received noninvasive mechanical ventilation at night. Patient 3 experienced with time symptoms of chronic hypoventilation like fatigue, diurnal headache, and loss of weight and was scheduled for evaluation of nocturnal oximetry and treatment. Patient 4 had an acute respiratory infection and died in few days after exacerbating respiratory distress with the chest X-ray showing bronchopneumonia, diaphragmatic paresis, and a severely reduced chest volume. Autopsy showed severe right ventricle hypertrophy consistent with a cor pulmonale, generalized severe reduction of muscle fibres that were mostly replaced by fibrotic tissue in the diaphragm and by fat and fibrosis in the other muscle groups [34]. These results are within the frame of the course of respiratory function in UCMD [7]. 

According to BMI (Table 3), 2 patients (3 and 6) remained in the underweight category at baseline and followup, one (patient 2) was in the healthy weight category at baseline and moved to the obese category at followup, one moved from the overweight to the obese (patient 5), and 2 patients (patient 1 and 4) remained in the obese category at baseline and followup. However, DXA (Table 3) showed that all the patients had already a high % of body fat at baseline, including the 2 underweight. In addition, during treatment, the lean mass decreased by 8.5%, while the fat mass increased by 37.6% in 5 patients (Table 3). The patients had a marked reduction of BMD irrespective of the level of motor function: walkers and nonwalkers had the same *Z* score mean value (−3.8). The BMD was not influenced by the treatment with CsA.

Two patients experienced hyrsutism (1 and 3), which was particularly evident in patient 1. None of the patients showed signs of renal dysfunction.

**Figure 1:**
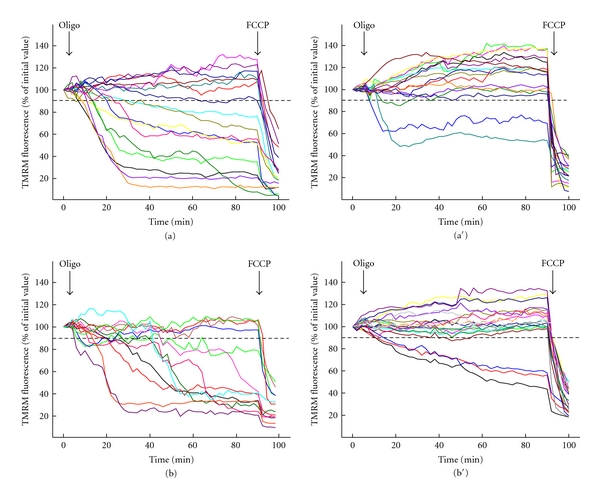
Changes of mitochondrial TMRM fluorescence induced by oligomycin in muscle cells isolated from patients before and after treatment with CsA. Muscle cells obtained from biopsies of two patients (P5 in a and a′, P6 in b and b′) were seeded onto glass coverslips precoated with laminin/poly-L-lysine, loaded with TMRM, and studied as described in Section 2. The procedure was repeated before (a, b) and after (a′, b′) 3 months of oral treatment with CsA. Where indicated, 6 *μ*M oligomycin (Oligo) and 4 *μ*M carbonylcyanide-*p*-trifluoromethoxyphenyl hydrazone (FCCP) were added. Each line represents one individual cell. Depolarized cells were 59% before (a) and 13% after (a′) CsA treatment in Patient 5, and 69% before (b) and 15% after (b′) CsA treatment in Patient 6.

In keeping with the results obtained in cultures from the first 3 UCMD patients [15] after 1 month of CsA treatment, the last 2 patients also showed that the mitochondrial membrane potential response to oligomycin was largely normalized in the muscle cells (Figure 1). If a threshold is arbitrarily set at 90% fluorescence, the number of cells with depolarizing mitochondria shifted from 89%, 91%, 95%, 59%, and 69% to 45%, 38%, 41%, 13%, and 15% before and after treatment with CsA for patients 1, 2, 3, 5, and 6, respectively (on average from 80.6% to 30.4%). These findings are in keeping with the results obtained in cultures from UCMD patients [14] and in fibers from *Col6a1^−/−^* mice [12], and they indicate that, at the dose used in this study, CsA reaches pharmacologically active concentrations in muscle. 

**Figure 2:**
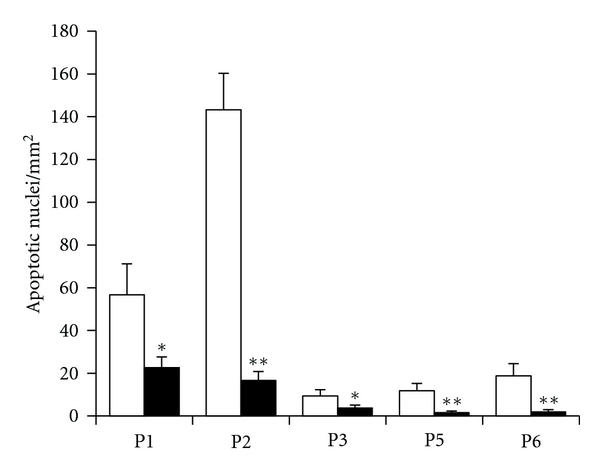
Apoptosis in muscle biopsies from UCMD patients before and after treatment with CsA. Biopsies from 5 patients with UCMD (P1–P3, P5, and P6) were scored for the presence of apoptotic nuclei with the TUNEL reaction as described in Section 2 before (open bars) and after 1 month (P1–P3) or 3 months (P5, P6) of oral treatment with 5 mg 7 kg CsA per day (filled bars). Data are expressed as mean ± SEM of three independent experiments. *P < 0.05. **P < 0.01. The incidence of apoptotic nuclei in healthy donor (control) biopsies was 0.39 ± 0.3 per square millimeter.

The treatment with CsA in patients 5 and 6, like in the first 3 UCMD patients [15], also showed a significant decrease of the occurrence of apoptosis (Figure 2). The incidence of apoptosis was assessed in the same bioptic materials used for the measurements of mitochondrial function. Unlike samples from healthy donors, where apoptosis was almost undetectable (0.39 ± 0.3 per square millimeter) [15], the samples from patients 5 and 6 had a number of apoptotic nuclei (15.3 ± 0.3 per square millimeter), which were considerably decreased after CsA treatment (1.8 ± 0.9 per square millimeter), indicating a cause-effect relationship between normalization of mitochondrial function and decreased cell death.

**Figure 3:**
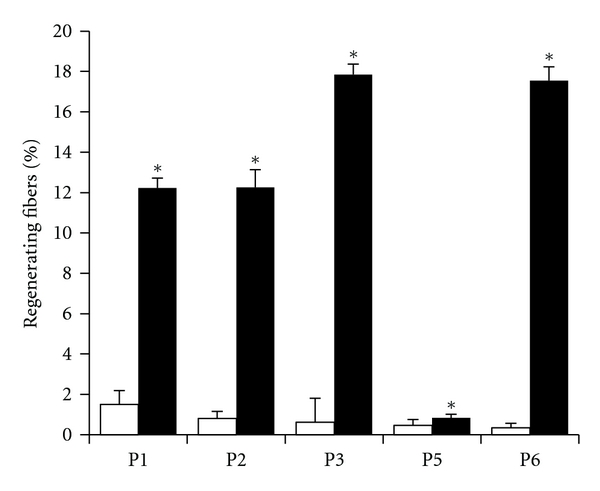
Regeneration in muscle biopsies from UCMD patients before and after treatment with CsA. Biopsies from 5 patients with UCMD (P1–P3, P5, and P6) were scored for the presence of regenerating fibers (labeled with antibody against developmental myosin heavy chain) as described in *Methods* before (open bars) and after 1 month (P1–P3) or 3 months (P5, P6) of oral treatment with 5 mg 7 kg CsA per day (filled bars). Data represent mean ± SD. * *P* < 0.01.

**Figure 4:**
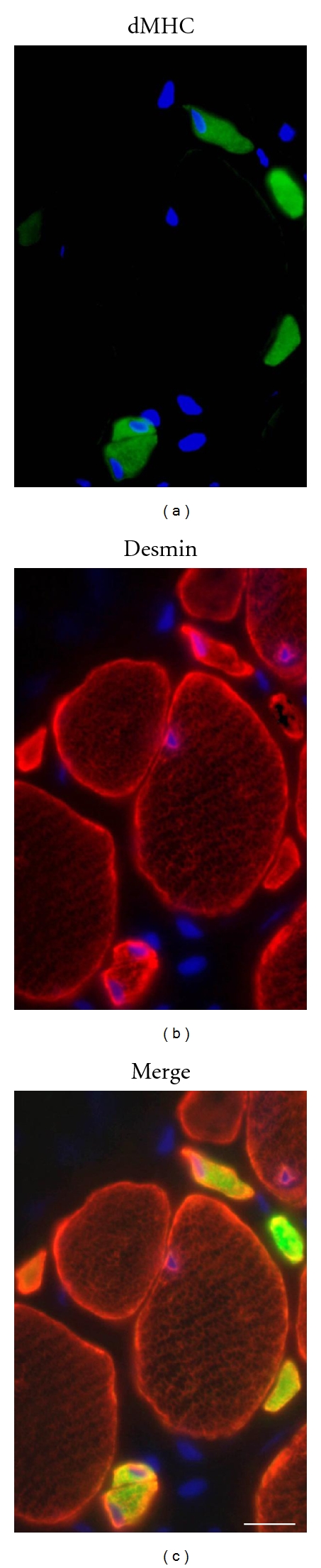
Regenerating muscle fibers. Representative cross-section of the muscle biopsy of patient 6 after 3-month treatment with CsA. The section was stained with DAPI to identify nuclei (blue) and with antibodies against developmental myosin heavy chain (dMHC, green), as marker of regeneration, and desmin (red), as general marker of muscle cells. Several very small calibered muscle fibers dMHC-positive, which also display an intense and diffuse staining for desmin, are detected among myofibers, pointing to an active regeneration process. Bar, 20 *μ*m.

After treatment with CsA, we found a statistically significant increased number of myofibers positive for developmental myosin heavy chain also displaying diffuse staining for desmin, which are characteristic signs of regeneration [27, 35]. The effect was particularly prominent in patients 1, 2, 3, and 6. Remarkably in patient 6, a marked regeneration was still evident in the biopsy taken after 3 months (Figures 3 and 4). The search for necrotic fibers with histochemical analysis of acid phosphatase activity and anti-C5b9 antibody was negative in all the biopsy taken before and after treatment with the exception of the baseline biopsy of patient 5 where a unique necrotic fiber was detected.

## 4. Discussion

In five out of the six children with UCMD, the prolonged CsA treatment resulted in an encouraging, statistically significant increase in the composite measure of muscle strength. However, it should be noted that the muscle strength improvement was not matched by a change in motor function. In contrast with the positive impact on limb muscle strength, CsA was not able to impede the progressive deterioration of the respiratory function. 

As we have already anticipated [15], the clinical effect of this treatment in attenuating progression of the disease and/or in translating into a better muscle performance depends on multiple factors, such as muscle loss and extent of fibrosis at the time of treatment, and potential for muscle regeneration in individual patients. Therefore, the discrepancy between the results of muscle function and muscle strength may have different explanations. Because in the muscular dystrophy functional grading scales there are only a few grades, improvement may not be noticed until a consistent change occurs [36, 37]. A cardinal measure of strength, like the isometric maximal voluntary contraction estimated with a dynamometer, may show the trend much sooner [36]. This is supported by the classic study of Bohannon [38] who evaluated the effect of resistant exercises in patients with amyotrophic lateral sclerosis. There were changes of 45 to 65 per cent in 14 of 18 muscles tested by hand-held dynamometry measurements, but no change in muscle graded strength. It should also be recognized that the response of a treatment may vary in different patients with the same disorder [37]. Given the progressive nature of UCMD, it was anticipated that the more effective the treatment is, the earlier it is started [37] when muscle substitution/degeneration is less severe. The youngest treated patient (patient 6) had in fact a good clinical result matched by a more diffuse regeneration in the muscle biopsy taken 3 months after treatment. It has also been suggested that a treatment would be more beneficial in patients with Bethlem myopathy, the milder form of the disease [34]. Another possibility is that different muscles may respond differently to CsA. The selective involvement of different muscles in the muscular dystrophies is a consistent finding that has repeatedly resisted explanation [39]. Gene expression studies may help explain why generalized genetic insults lead to consistent patterns of weakness that affect some muscles more than others [39] and why the same treatment has different impacts on different muscles in the same patient [40]. In particular, the differential effect of CsA on limb versus respiratory muscle strength could be related to the grade of muscle involvement, with the more severely affected respiratory muscle being less prone to respond. The diaphragm in fact is the most severely compromised muscle in the otherwise mildly affected *Col6a1^−/−^* mice [11]. The diaphragm is also invariably affected in UCMD patients, causing early and severe respiratory insufficiency requiring respiratory support in the first or second decade of life [7, 10]. A generalized severe reduction of muscle fibres that were mostly replaced by fibrotic tissue was found in the diaphragm at autopsy of the patient who died after bronchopneumonia [34]. A rapid progression of respiratory insufficiency has been consistently recognized in UCMD patients [6, 10, 37]. A review of the course of respiratory insufficiency in 13 patients with UCMD showed that FVC has been found abnormal in all patients from age 6 with a yearly decline (% predicted) of 6.6% from age 6 to 10 [7], 69.2% of patients were already on noninvasive nocturnal ventilation, and 2 patients have died of respiratory insufficiency [7]. Apart from the 6 UCMD patients in this study, 4 younger of 6 years and not able to perform spirometry, we have followed other 4 patients, all of them had a progressive decline of FVC, one died at 11 years during an intercurrent respiratory illness, and the other 3 started mechanical ventilation at a mean age of 11.1 (SD 2.8). 

In our patients, the evaluation of body composition gave some contrasting results between BMI and DXA. With the DXA, all the patients had a marked excess of fat mass in their body weight. The body fat was 58% of body weight at baseline (range 31–74) and reached 72.2% at followup (range 53%–80%). However, the weight status for the calculated BMI-for-age percentile was for one of these patients “healthy weight” and for 2 patients “underweight”. It is clear that because BMI does not differentiate fat from muscle mass, it is not the proper way to evaluate the body composition of dystrophic patients. In addition, DXA clearly documented the complete and progressive overturn of body composition in UCMD patients with % body fat increasing from 60.4 to 72.6 during the treatment period compared to less than 30% of body fat in normal children [23]. BMD was markedly decreased in UCMD patients compared with healthy matched controls, and these differences in BMD values were not correlated with the ambulatory status nor with increasing age as it was found in Duchenne muscular dystrophy patients [41].

The principal adverse reactions associated with the use of CsA are renal dysfunction, hypertension, headache, gastrointestinal disturbances, and hyrsutism/hypertrichosis. Two of our patients had hyrsutism/hypertrichosis, which was more evident in patient 1, a female. 

The biological results of this study confirm the previous ones [15] and prove that treatment with moderate doses of CsA favourably affects mitochondrial function in collagen VI myopathies *in vivo *and dramatically decreases the frequency of muscle cell death in the patients. Muscle fiber necrosis was almost absent in the muscle biopsies of the UCMD patients both before and after CsA treatment, confirming that in UCMD there are scarce necrotic fibers [35, 42]. In the last two patients like in the first 3 [15], we found that regeneration was very limited at baseline, but significantly increased after CsA treatment for 3 and 1 month, respectively. Interestingly, a peculiar pattern of regeneration was found in 2 patients with advanced stage of UCMD [35] and in patients with Bethlem myopathy [42]. The Japanese authors [35], on the basis of the absent expression of developmental myosin heavy chain in regenerating very small calibered muscle fibers, suggested that collagen VI deficiency may disturb not only the mechanical integrity of skeletal muscle fibers but also ongoing regeneration or complete maturation of muscle fibers at least in the advanced stage of UCMD. They also anticipated that it would be important to determine if the lack of expression of developmental myosin heavy chain is common to all patients with UCMD, in particular in patients at early stages of the disease [35]. Our findings show that younger patients with UCMD express indeed developmental heavy chain in the small regenerating fibers and that the number of the very small calibered regenerating fibers expressing developmental heavy chain and desmin is significantly increased compared to baseline after CsA treatment. Our data suggest that in younger patients with UCMD, the abnormal regeneration or maturation processes are less affected as compared to the situation in older patients, and that CsA treatment is effective in correcting the abnormality. At first sight, the observation that treatment with CsA increased regeneration may appear paradoxical because regeneration should match the rate of muscle fiber death, which is lower when CsA is present. An appealing explanation [15] is that in collagen VI diseases, differentiating muscle cells are undergoing early apoptosis together with mature fibers, a situation that would be similar to the ineffective erythropoiesis seen in many hemoglobinopathies. By decreasing ineffective myogenesis, CsA could increase the overall efficiency of muscle regeneration, in keeping with the response of all patients.

The potential benefit of CsA in UCMD patients must be carefully weighed against the possible risks of immunosuppression. It should be mentioned, however, that other cyclosporins, like MeAla3EtVal4-cyclosporin (Debio 025), may represent an alternative to CsA [43]. We have shown that Debio 025, a nonimmunosuppressive cyclosporin that retains the PTP-desensitizing properties of CsA, is as effective as CsA at protecting mitochondrial function and preventing apoptosis in muscle cultures from UCMD patients [14], a finding that holds great promise for the treatment of collagen VI disorders.

After the completion of this trial, we have shown that muscle pathology in *Col6a1^−/−^* mice lacking collagen VI is ameliorated by a low-protein diet and by genetic and pharmacologic approaches, including CsA, that activate autophagy [44, 45]. The present as well as previous studies [12, 14, 15, 44] are moving from the animal model of collagen VI myopathy and its correction with drugs to the confirmation of the same pathogenesis and disease correction in patients with Bethlem myopathy and UCMD.

## 5. Conclusions

Clarification of the pathogenic mechanisms of muscle fiber damage in the animal model of collagen VI muscular dystrophies is allowing the rapid transfer to man of experimental therapies based on inhibition of mitochondrial dysfunction with CsA. The present demonstration that long-term treatment with CsA ameliorates performance in specific muscles of 5 out 6 UCMD patients and improves regeneration is an encouraging result that raises some hope for the use of cyclophilin inhibitors for the cure of this and possibly other muscle diseases.

## Figures and Tables

**Table 1:** Patient's details. Phenotype, motor function, collagen VI type of expression in muscle or skin biopsies, and mutation in *COL6A* genes are reported for each patient. All the patients had a UCMD clinical phenotype. At baseline, P5 and P6 were able to walk. ColVI levels are based on immunoistochemistry as described in original references [2, 14]. Patients had mutations in each of the 3 *COL6A* genes both *de novo* or compound heterozygous.

Patient	Phenotype	Collagen VI	Mutation(s)
1	UCMD, NW	Mild reduction in muscle fibers and fibroblasts	*COL6A1 de novo *heterozygous Gly284Arg [14]
2	UCMD, NW	Marked reduction in muscle fibers	*COL6A2 *compound heterozygous Gly487-Ala495delAspfsX48 and Glu591-Cys605delThrfsX148 [2]
3	UCMD, NW	Marked reduction in muscle fibers	*COL6A1 de novo *heterozygous del275-280/insGlu275 [14]
4	UCMD, NW	ND	*COL6A2 *compound heterozygous Gly487-Ala495delAspfsX48 and Glu591-Cys605delThrfsX148 [2]
5	UCMD, W	Moderate reduction in muscle fibers	*COL6A2* compound heterozygous intron 8 c.927 + 5 G>A het p.Lys318fsX6
6	UCMD, W	Moderate reduction in muscle fibers	*COL6A3* heterozygous G to A nt 6465 +1

UCMD: Ullrich congenital muscular dystrophy; W: walker; NW: nonwalker; ND: not done.

**Table 2:** Study results. The three patients enrolled in the original pilot trial (P1, P2, and P3) received CsA for 2 to 3.2 years and the three new patients for 1 to 1.2 years. Megalimbs increased in all but P5. The distance walked in 6 minutes remained the same in the 2 patients able to walk. Forced vital capacity declined in all from 57.1% predicted to 45.2% predicted.

Patient	Age at treatment (Years)	Age at Followup (Years)	Megalimbs (Newton)		6 MWD (meter)		FVC %	
			Time 0	Followup	Time 0	Followup	Time 0	Followup
1	9.5	12.7	218	283	NA	NA	47	24
2	9.8	11.6	106	142	NA	NA	44	32
3	8.9	10.9	106	120	NA	NA	NA	NA
4	9.0	10.2	140	169	NA	NA	41.7	31
5	9.1	10.1	241	222	189	188	59	51
6	5.5	6.7	172	236	300	304	94	88

Mean	8.6	10.4	163.8	195.3	244.5	246	57.1	45.2

Megalimbs: sum of bilateral muscle strength of elbow flexion, hand grip, knee flexion, and knee extension; 6 MWD: distance walked in 6 minutes; FVC %, forced vital capacity % of predicted; NA: not able.

**Table 3:** Body composition evaluation: BMI and DXA. Body fat was estimated with the body mass index (BMI) and DXA. The BMI number using the CDC BMI-for-age growth charts for girls and boys is translated into a percentile that categorizes the weight status. Two patients (P3 and P6) remained in the underweight category during the study, one moved from the overweight to the obese (P 5), and 2 patients (P1 and P4) remained in the obese category at baseline and followup. However, all the patients had already a high % of body fat at baseline, including the 2 underweight. In addition, the lean mass decreased by 8.5%, while the fat mass increased by 37.6% during the treatment in 5 patients. The patients had a marked reduction of total body BMD irrespective of the level of motor function: walkers and nonwalkers had the same *Z* score mean value (−3.8). The BMD was also not influenced by the treatment with CsA.

Patient			DXA							
	BMI (kg/m^2^)		Lean Tissue Mass (kg)		Fat Tissue Mass (kg)		FAT%		BMD *Z* score	
	Time 0	Followup	Time 0	Followup	Time 0	Followup	Time 0	Followup	Time 0	Followup
1	29.8	27.3	12.2	ND	40.7	ND	74	ND	−4.3	ND
2	15.5	23.2	9.8	10.8	18.9	40.2	63	77	−3.1	−3.9
3	13.9	12.1	8.7	ND	10.1	ND	52	ND	−4.1	ND
4	23.8	27.6	12.9	12.5	31.8	55.5	68	80	−3.6	−3.7
5	20.4	22.4	8.6	7.1	22.8	30.7	70	79	−4.6	−3.3
6	11.1	13.2	8.8	6.6	4.1	8.1	31	53	−3.1	−3.6

Mean	19.1	21.2	10.0*	9.2*	19.4*	33.6*	58*	72.2*	−3.6*	−3.6*

BMI: body mass index; DXA: dual-energy X-ray absorptiometry; BMD: bone mineral density (g/cm^2^); ND: not done; *: mean of DXA data from patients 2, 4, 5, 6.
